# Utilizing urinary single-cell RNA sequencing to explore the pathogenesis of diabetic kidney disease progression

**DOI:** 10.1093/ckj/sfag230

**Published:** 2026-07-10

**Authors:** Henry H L Wu, Long The Nguyen, Naveen Kumar Parthiban, Joey Lai, David Zheng, Chia-Ling Chan, Robert J Walker, Carol A Pollock, Sonia Saad

**Affiliations:** Renal Research, Kolling Institute of Medical Research, Royal North Shore Hospital & The University of Sydney, Sydney, Australia; Department of Renal Medicine, Royal North Shore Hospital, Northern Sydney Local Health District, Sydney, Australia; Renal Research, Kolling Institute of Medical Research, Royal North Shore Hospital & The University of Sydney, Sydney, Australia; Renal Research, Kolling Institute of Medical Research, Royal North Shore Hospital & The University of Sydney, Sydney, Australia; Westmead Genomics Facility, Westmead Institute of Medical Research, Westmead Hospital & The University of Sydney, Sydney, Australia; Westmead Genomics Facility, Westmead Institute of Medical Research, Westmead Hospital & The University of Sydney, Sydney, Australia; Westmead Genomics Facility, Westmead Institute of Medical Research, Westmead Hospital & The University of Sydney, Sydney, Australia; Department of Medicine (Dunedin), Faculty of Medicine, The University of Otago, Dunedin, New Zealand; Renal Research, Kolling Institute of Medical Research, Royal North Shore Hospital & The University of Sydney, Sydney, Australia; Department of Renal Medicine, Royal North Shore Hospital, Northern Sydney Local Health District, Sydney, Australia; Renal Research, Kolling Institute of Medical Research, Royal North Shore Hospital & The University of Sydney, Sydney, Australia

To the Editor,

Determining the risk of progression to end-stage kidney disease from early diabetic kidney disease (DKD) is important for optimization of therapy. The availability of single-cell ribonucleic acid sequencing (scRNAseq) technology allows for measurement of gene expression at a single-cell level and provides detailed insights on subpopulations of cells in kidney disease and their roles in disease progression [1]. There is now recognized potential in utilizing scRNAseq as a prognostic biomarker in early DKD, with urinary exfoliated kidney cells being previously applied as noninvasive biomarkers for DKD prognostication [2]. Whilst the pathogenesis of DKD was previously explored using serum scRNAseq, few studies have done so currently based on urinary scRNAseq [3–5]. There were no published studies which considered the utility of urinary scRNAseq to determine the pathogenesis of DKD progression over time. Our group conducted a pilot study to explore on this.

**Figure 1:**
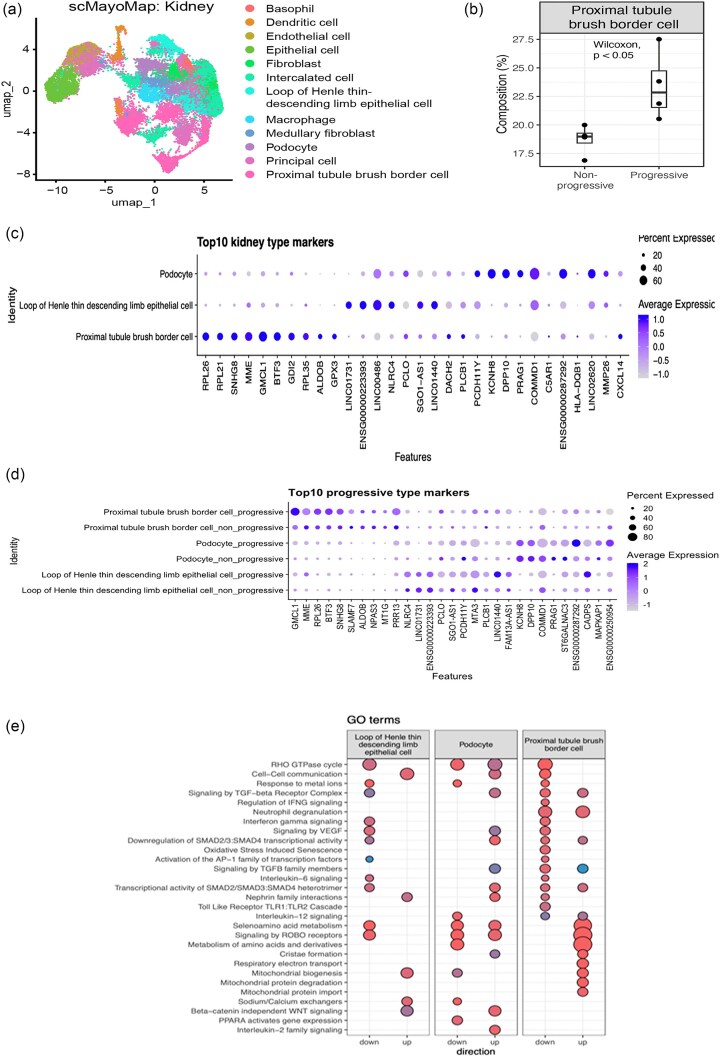
Comparison between the ‘progressive’ versus ‘nonprogressive’ DKD groups by urinary scRNAseq analysis. (A) Distribution of specific cells identified in urine of patients with DKD in our pilot study cohort (*n* = 8) based on the single-cell MayoMap software (hematopoietic, kidney, and epithelial cells were detected in significant amounts for analysis). (B) Percentage of urinary exfoliated PTCs with respect to other cell types in urine of patients with ‘progressive’ (*n* = 4) versus ‘nonprogressive’ (*n* = 4) DKD, *P *< .05. (C) Top 10 genes expressed in urinary exfoliated podocytes, loop of Henle, and PTCs between the ‘progressive’ (*n* = 4) and ‘nonprogressive’ (*n* = 4) DKD groups. (D) Top 10 genes that are significantly regulated in urinary exfoliated podocytes, loop of Henle, and PTCs between the ‘progressive’ (*n* = 4) and ‘nonprogressive’ (*n* = 4) DKD groups. (E) Signaling pathways which are highly regulated in urinary exfoliated podocytes, loop of Henle, and PTCs between patients with ‘progressive’ (*n* = 4) and ‘nonprogressive’ (*n* = 4) DKD (colors reflect *P* values; upregulated and downregulated genes are shown; circle size represents gene ratio between the ‘progressive’ and ‘nonprogressive’ DKD groups).

Eight adults with diabetes mellitus and early-stage DKD at study baseline were recruited. Early-stage disease was defined as estimated glomerular filtration rate (eGFR) 60–90 ml/min/1.73 m^2^. Four individuals were classified in the ‘progressive’ DKD group and four individuals were classified in the ‘nonprogressive’ DKD group. ‘Progressive’ DKD here is defined by eGFR >5 ml/min/1.73 m^2^/year over 3-year follow-up and worsened albuminuria status (i.e. normoalbuminuria to microalbuminuria or macroalbuminuria; or microalbuminuria to macroalbuminuria) in an individual with diabetes mellitus. ‘Nonprogressive’ DKD is defined by eGFR ≤5 ml/min/1.73 m^2^/year over 3-year follow-up with no change or improved albuminuria status (i.e. no change in albuminuria status; macroalbuminuria to microalbuminuria or normoalbuminuria; or microalbuminuria to normoalbuminuria) in an individual with diabetes mellitus. In each group, there were two males and two females, respectively. No statistically significant differences in age between the groups were identified (mean 59.6 vs 56.0 years, *P *= .766). Here, 800–1000 ml of urine were collected from each individual at baseline. Urinary exfoliated kidney cells in each sample were washed, collected, labelled with barcoding antibodies (BD Rhapsody Enhanced Cartridge Reagent Kit), and stained with viability dyes (Calcein AM & Draq7). The same number of viable cells from each sample were pooled and sorted by an INCYTO hemocytometer, after which the pooled samples were subjected to the BD Rhapsody Single Cell Analysis System followed by standard bioinformatics analyses. Numerous kidney, hematopoietic, and epithelial cell types were identified in significant amounts from urine of the eight DKD patients (Fig. 1A). Proximal tubule cells (PTCs) were the predominant cell type amongst the urinary exfoliated kidney cells, accounting for >20% of the cell population on average. Individuals in the ‘progressive’ DKD group exfoliated significantly more PTCs compared to those in the ‘nonprogressive’ DKD group (Fig. 1B). scRNAseq analysis of the urinary cells elucidated genes which were specifically expressed in the different cell types, as well as genes which were significantly regulated in these cells between the ‘progressive’ versus ‘nonprogressive’ DKD groups. The top 10 genes expressed (Fig. 1C) and significantly regulated (Fig. 1D) between the ‘progressive’ versus ‘nonprogressive’ DKD groups in urinary podocytes, loop of Henle, and PTCs are shown. Signaling pathways that are significantly regulated between the two groups were identified (Fig. 1E). These included mitochondrial and oxidative stress pathways, fibrosis pathways (transforming growth factor-β and SMAD signaling pathways), inflammation pathways (interferon-γ, interleukin-6, and activator protein-1 that modulate nuclear factor kappa-light-chain-enhancer of activated B cells activity), and metabolic pathways (peroxisome proliferator-activated receptors signaling and toll-like receptors).

Evaluating the above findings, our pilot data strongly suggest that scRNAseq analysis of urinary exfoliated cells may provide a noninvasive, unprecedented insight into the cellular processes that underlie DKD progression. Further study is now required to correlate the changes in molecular signatures over time in urinary exfoliated cells to treatment response and select individuals likely to benefit from subsequent therapeutic intervention.
